# Deep learning (DL)‐based advancements in prostate cancer imaging: Artificial intelligence (AI)‐based segmentation of ^68^Ga‐PSMSA PET for tumor volume assessment

**DOI:** 10.1002/pro6.70014

**Published:** 2025-05-03

**Authors:** Sharjeel Usmani, Khulood Al Riyami, Subash Kheruka, Shah P Numani, Rashid al Sukaiti, Maria Ahmed, Nadeem Pervez

**Affiliations:** ^1^ Department of Radiology and Nuclear Medicine Sultan Qaboos Comprehensive Cancer Care and Research Centre (SQCCCRC) University Medical city Muscat Oman; ^2^ Department of Radiology Carl T Hayden VA Medical Center Phoenix USA; ^3^ Medical University of Lublin Lublin Poland; ^4^ Department of Radiation Oncology Sultan Qaboos Comprehensive Cancer Care and Research Centre (SQCCCRC) University Medical city Muscat Oman; ^5^ Internal Medicine College of Medicine and Health Sciences UAE University Abu Dhabi United Arab Emirates

**Keywords:** ^68^Ga‐PSMA, PET‐CT, prostate cancer, artificial intelligence, deep learning, radiotherapy, radiation treatment

## Abstract

Positron emission tomography (PET) with gallium‐68 prostate‐specific membrane antigen (^68^Ga‐PSMA) has emerged as a promising imaging modality for evaluating prostate cancer (PC). Quantification of tumor volume is crucial for staging, radiotherapy treatment planning, response assessment, and prognosis in PC patients. This review provides an overview of the current methods and challenges in the assessment of regional and total tumor volumes using ^68^Ga‐PSMA PET. Traditional manual segmentation methods are time‐consuming processes that are further challenged by inter‐observer variability. Artificial intelligence (AI)‐based segmentation techniques offer a promising solution to these challenges. AI algorithms, such as deep learning‐based models, have shown remarkable performance in automating tumor segmentation tasks with high accuracy and efficiency. This review discusses the principles underlying AI‐based segmentation algorithms, including convolutional neural networks, and their applications in PC imaging. Furthermore, the advantages of AI‐based segmentation are highlighted, such as improved reproducibility, faster processing times, and potential for personalized medicine. Despite these advancements, AI‐based segmentation faces significant challenges, including the need for standardized protocols, extensive validation studies, and seamless integration into clinical workflows. Addressing these limitations is essential for the widespread adoption of AI‐based segmentation in ^68^Ga‐PSMA PET for PC, ultimately advancing the field and improving patient care.

## Graphical abstract

This review highlights the use of 68Ga‐PSMA PET in prostate cancer (PC) for tumor volume quantification, crucial for staging, treatment planning, and prognosis. Traditional manual segmentation methods are time‐consuming and variable, while AI‐based segmentation techniques, particularly deep learning models like convolutional neural networks, offer automated, accurate, and efficient solutions. AI‐based segmentation improves reproducibility and processing times, aiding personalized medicine. However, challenges such as standardized protocols, extensive validation, and clinical integration need addressing for its widespread adoption in 68Ga‐PSMA PET for PC, enhancing patient care.

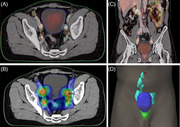

## 1 INTRODUCTION

Prostate cancer (PC) is the second most common cancer and the fifth most common cause of cancer‐related deaths in men.^1^ Recent advancements have improved treatment pathways for primary localized disease, locally advanced disease, oligometastatic cancer, and low‐ and high‐volume metastatic diseases.^2^ Gallium‐68 prostate‐specific membrane antigen (^68^Ga‐PSMA) is a proven tracer for both the staging and detection of biochemical recurrence (BCR) in PC and is superior to conventional imaging modalities.^3^, ^4^ Prostate‐specific membrane antigen (PSMA), a membrane‐specific type II glycoprotein, is over expressed in more than 80% of PC cells and is therefore an ideal target for diagnostic imaging.^5^, ^6^ The detection rate of PSMA‐positron emission tomography‐computed tomography (PET‐CT) reported in the literature varies from 46% to 97%, depending on the prostate‐specific antigen (PSA) levels.^7^ Remarkably, PC can be detected even at very low PSA levels, with a detection rate of approximately 40% in patients with PSA level below 0.5 ng/mL.^8^ Compared with other imaging modalities, ^68^Ga‐PSMA PET surpasses multi‐parametric magnetic resonance imaging (mpMRI) in terms of sensitivity for detecting lesions, particularly in recurrent disease and low PSA settings. It also offers advantages over fluorine‐18 (^18^F)‐fluciclovine PET, which is effective for detecting recurrent PC but is less sensitive for low‐volume disease. ^68^Ga‐PSMA PET provides comprehensive insights into the disease's total extent and tumor burden, offering a prime opportunity to investigate the role of targeted therapies.

Artificial intelligence (AI) and deep learning (DL) have further revolutionized nuclear medicine and molecular imaging. DL‐based methods for automated segmentation of soft tissue and bone enhance the detection of nodal, soft tissue, and bone metastases in ^68^Ga‐PSMA PET‐CT. Automated biomarker analysis determines tumor burden by calculating the mean standardized uptake value (SUV) in volumes of interest, such as the prostate gland, lymph nodes, and bones.^9^ These advancements highlight the integration of AI‐driven innovations in improving diagnostic precision and supporting personalized treatment strategies for PC.

## 2 ^68^Ga‐PSMA PET‐CT

### 2.1 Principles of ^68^Ga‐PSMA PET

^68^Ga‐PSMA is a promising tracer for staging at initial diagnosis and for BCR in PC.^10^ PSMA is a type‐2 transmembrane glycoprotein, alternatively referred to as glutamate carboxypeptidase II or N‐acetyl‐L‐aspartyl‐L‐glutamate peptidase I, and is highly expressed in PC.^10^, ^11^ This protein is endogenously found in several body tissues, such as prostate epithelium, salivary and lacrimal glands, certain brain tissues, segments of the digestive system (the duodenum and colon), and proximal tubules in the kidneys.^11^ PSMA is significantly upregulated at both primary organ and metastatic sites in PC. In addition to PC, PSMA expression is observed in many other tumors, and their metastases are often associated with neovascularization. Furthermore, PSMA can be identified in certain non‐malignant disorders characterized by granulomatous or inflammatory reactions.^10^ PSMA‐targeting tracers are used in diagnostic imaging modalities such as single‐photon emission computed tomography (SPECT/CT) and PET‐CT. These tracers can be marked with various isotopes, including technetium‐99m (Tc‐99m), ^68^Ga, and ^18^F, based on specific diagnostic requirements. Tracers may be ligands with isotopes such as lutetium‐177 (^177^Lu) or actinium‐225 (^225^Ac) for theragnostic purposes, including both therapy and diagnosis.^12^

When ^68^Ga‐labeled tracers are not accessible, Tc‐99m‐labeled PSMA tracers are often used.^13^ Although many radiopharmaceuticals are used, no conclusive data indicate that any radiopharmaceutical provides higher diagnostic precision than others. This may be ascribed to the small variations among these tracers. For example, the isotope ^68^Ga has a half‐life of 68 minutes and is usually generated from a germanium‐gallium generator. In contrast, the isotope ^18^F, which has a half‐life of 120 minutes, does not require the presence of a cyclotron at the site. PSMA PET tracers are physiologically distributed in the lacrimal gland, salivary glands, kidneys, ureters, and bladder. Further, modest activity has been observed in the duodenum, small intestine, liver, and spleen.

### 2.2 Protocol of ^68^Ga‐PSMSA PET

In terms of imaging techniques, the usual range of activity for ^68^Ga‐PSMA‐11 is 111–259 MBq (3–7 mCi), with a suggested uptake time of 50 to 100 minutes. For ^18^F‐DCFPyL, the normal range of activity is 296–370 MBq (8–10 mCi), with an uptake time of 60 minutes. Delayed imaging may be considered an alternative in situations with elevated bladder urine activity. PSMA PET may be performed in combination with either CT (PET‐CT) or MRI (PET/MRI).^14^

### 2.3 Clinical advantage and literature review

Multiple studies suggest that the use of ^68^Ga‐PSMA PET‐CT, either alone or in combination with mpMRI, improves the identification of clinically relevant PC.^15^ PSMA PET‐CT‐guided biopsies have shown greater accuracy than mpMRI in detecting multifocal and bilateral illness in the treatment setting.^16^ Research has highlighted the efficacy of PSMA PET‐CT in managing BCR, especially when dealing with low PSA levels.^8^, ^12^ This suggests that PSMA PET‐CT has the potential to enhance the identification of metastasis, even at low PSA levels. PSMA expression in metastatic lesions paves the way for PSMA radioligand therapies, such as β/α emitter ^177^Lu/^225^Ac PSMA therapy, in metastatic PC.^17^, ^18^ However, certain limitations of ^68^Ga‐PSMA PET need to be acknowledged compared to those of other PSMA tracers, such as ^18^F‐PSMA. The shorter half‐life of ^68^Ga (approximately 68 minutes) restricts its availability to centers with on‐site generators, whereas ^18^F‐PSMA tracers, which have a longer half‐life (approximately 110 minutes), are more widely distributed. Additionally, ^18^F‐PSMA tracers, such as ^18^F‐PSMA‐1007, often provide sharper imaging resolution and lower background noise, thus enhancing lesion detection in certain regions such as the prostate bed or bones. Moreover, reduced urinary excretion of some ^18^F‐PSMA tracers makes them advantageous for detecting lesions near the bladder. Despite these differences, ^68^Ga‐PSMA PET remains highly effective in detecting BCR at extremely low PSA levels, demonstrating its critical role in PC management. Future studies exploring the complementary use of ^68^Ga and ^18^F tracers may further optimize PSMA‐targeted imaging strategies. Indications for ^68^Ga‐PSMAPET‐CT are listed in Table 1.

**TABLE 1:** Clinical indications for ^68^Ga‐PSMAPET‐CT in prostate cancer.

Indications for ^68^Ga‐PSMA PET‐CT
Initial staging of localized intermediate to high risk and metastatic prostate cancer Initial staging of younger patients Improve target delineation for radiation treatment by detecting metabolic lesion(s) and reducing inter‐observer variability Treatment response assessment of radiation or systemic treatments Assessment of biochemical recurrence post‐treatment Evaluation for possibility of PSMA radioligand therapies Targeted biopsy after previous negative biopsy in patients with high suspicion of prostate cancer

### 2.4 Radiotherapy and^68^Ga‐PSMA PET

**FIGURE 1:**
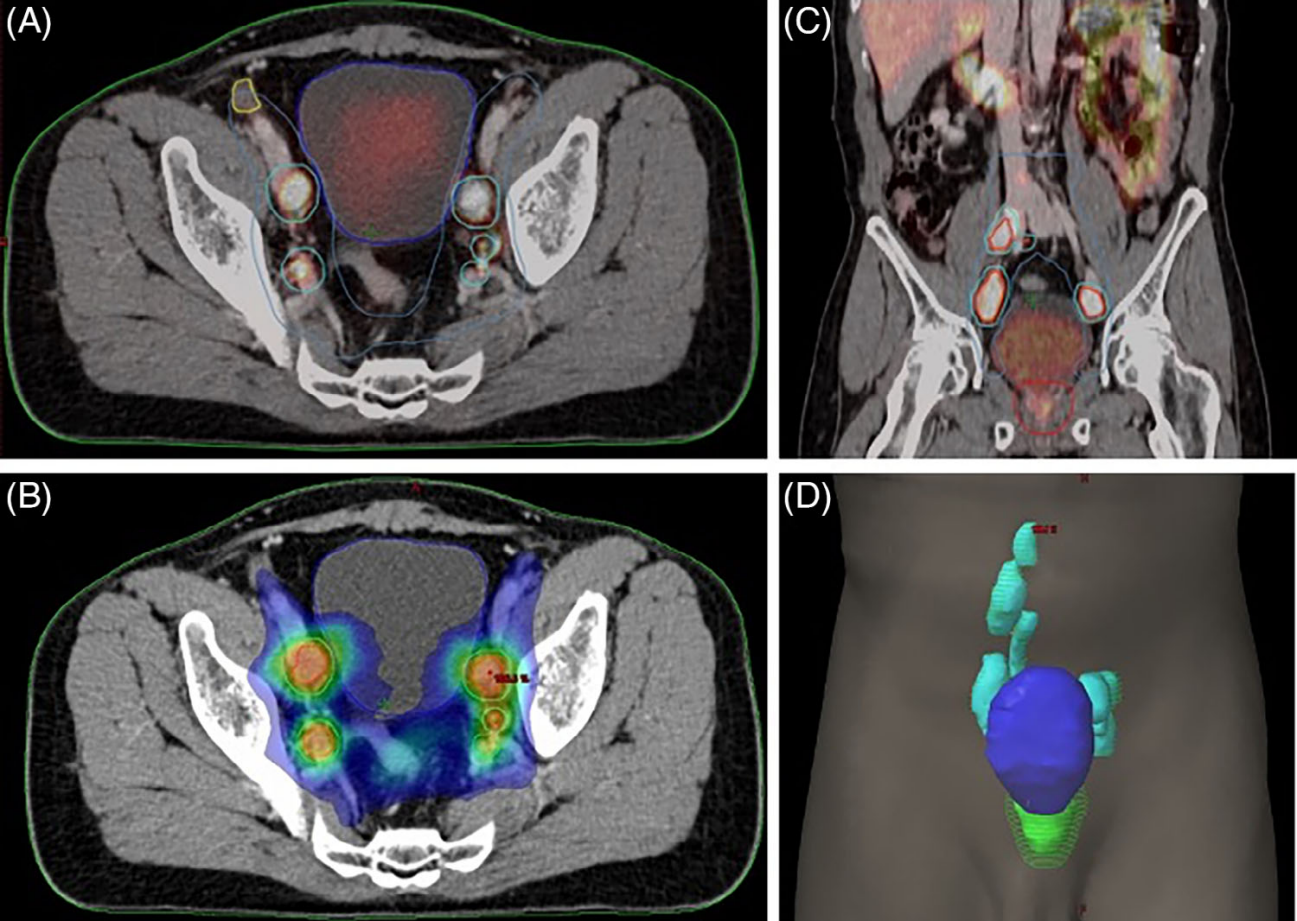
Deep learning‐based tumor delineation on 68Ga‐PSMA PET for treatment planning. (A and C) Single axial and coronal PSMA PET‐CT radiotherapy simulation images showing grossly involved avid pelvic lymph nodes from prostate cancer, marked by cyan color drawing for radiation treatment planning. Prostate gland is marked by red while bladder is marked by blue color. (B) Same image as shown in Figure 1(A) with radiation treatment planning. Red cloud shows high radiation doses for gross disease while blue cloud shows lower radiation doses to potential sites of micro‐metastasis. (D) Coronal view of body showing (i) prostate gland – green color volume, (ii) gross pelvic and para‐aortic lymph nodes – cyan color volume, and (iii) urinary bladder – blue color volume.

Tumor target volume delineation and treatment planning are integral components of radiation treatment. ^68^Ga‐PSMA PET‐CT significantly affects radiation planning by identifying metabolically active disease(s) that may not be detected by conventional anatomical imaging.^19^, ^20^, ^21^ In the context of radiotherapy, DL‐based tumor volume assessment using ^68^Ga‐PSMA PET plays an important role in target volume delineation and treatment planning (Figure 1). The ^68^Ga‐PSMA PET‐based segmented tumor volumes, obtained from DL analysis, are incorporated into radiotherapy planning processes. AI‐derived contours ensure better dose targeting to metabolically active regions while minimizing unnecessary radiation to healthy tissues, such as those of the bladder and rectum. This precision reduces the risk of acute and long‐term toxicity and improves patient outcomes. Additionally, DL‐based algorithms detect and segment tumor volumes with higher precision, reducing inter‐observer variability and improving consistency across cases.

AI models can also manage temporal changes in imaging data, such as tumor growth or shrinkage over time. By analyzing sequential imaging datasets, these models can detect changes in tumor volume or tracer uptake between pre‐ and post‐treatment scans. This functionality allows clinicians to quantify treatment response, identify residual or recurrent disease, and make data‐driven adjustments to therapy plans. Furthermore, their integration with adaptive radiotherapy workflows ensures real‐time updates to treatment plans based on observed anatomical or metabolic changes, thus maintaining dosing accuracy throughout therapy. The efficiency of AI tools also streamlines workflows by reducing the time required for tumor delineation, allowing clinicians to focus on optimizing treatment strategies. Integrating DL–based tumor volume assessment using ^68^Ga‐PSMA PET into radiotherapy planning enhances the precision, efficiency, and personalization of prostate cancer treatment. This approach enables more accurate delineation of tumor boundaries, facilitating tailored radiation strategies that improve therapeutic outcomes.

## 3 ARTIFICIAL INTELLIGENCE INTEGRATION IN PROSTATE CANCER IMAGING

The amalgamation of imaging data with genomic and molecular data signifies substantial progress in precision oncology, providing more comprehensive insights into tumor biology. AI can integrate these modalities, facilitating the extraction of complementary insights to improve diagnostic, prognostic, and therapeutic approaches.

### 3.1 Radiogenomics

Radiogenomics models powered by AI can connect the features of a ^68^Ga‐PSMA PET‐CT scan with genetic problems, such as androgen receptor mutations, TMPRSS2‐ERG fusions, or Phosphatase and Tensin Homolog (PTEN) loss, which is common in PC. These links facilitate the identification of imaging biomarkers that show how biological processes work at the molecular level. This could lead to noninvasive alternatives to tissue samples for judging how different and aggressive a tumor is.

### 3.2 Genomic Profile Prediction

AI algorithms can evaluate imaging data to forecast molecular phenotypes, including gene expression profiles and mutational loads. Machine learning algorithms can connect tracer uptake patterns to different genetic subtypes. This makes it easier to sort patients into groups for targeted medicine or clinical trials.

### 3.3 Treatment Personalization

The integration of imaging and genomic data with AI can enhance treatment options by recognizing molecular subgroups with unique imaging manifestations. Based on imaging results and molecular profiles, this method may help doctors choose better systemic drugs, such as androgen deprivation therapy or PSMA radioligand therapy.

### 3.4 Longitudinal Monitoring

AI may provide continuous integration of imaging and genomic data to monitor treatment responses and identify resistance mechanisms. Integrating temporal variations in ^68^Ga‐PSMA PET‐CT results with sequential genomic profiling may reveal adaptive tumor evolution and inform therapeutic modifications.

### 3.5 Multi‐Omics Approaches

In addition to genomics, the use of AI frameworks to combine imaging data with transcriptomics, proteomics, and metabolomics can provide new information on how tumors work. This multi‐omics integration may augment biomarker identification and deepen our understanding of PC progression.

### 3.6 Challenges in AI and Genomic Integration

Integrating imaging and genomic data, albeit promising, necessitates overcoming obstacles, such as aligning data across varying spatial and molecular dimensions, ensuring data quality and standardization, and developing AI models adept at managing multimodal datasets. To successfully train and evaluate these algorithms, future initiatives must prioritize the creation of extensively annotated datasets that include imaging and genomic data. By integrating imaging and genomics, AI can revolutionize PC management and advance personalized therapies that incorporate molecular and phenotypic insights into clinical decision‐making.

## 4 POTENTIAL APPLICATIONS OF DL IN ^68^Ga‐PSMA PET‐CT IMAGING

The introduction of ^68^Ga‐PSMA PET‐CT imaging, in conjunction with advanced DL analysis, presents significant advancements in the management of PC. In addition to diagnosis and staging, AI‐based segmentation can influence patient outcomes in several ways.

### 4.1 Enhanced Survival Rates

AI‐driven segmentation facilitates more accurate delineation of tumor volumes, resulting in improved targeting of radiotherapy and systemic therapies. Accurate identification of high‐risk areas and reduction in exposure to healthy tissues may enhance treatment efficacy and improve long‐term survival rates.

### 4.2 Improved Quality of Life

AI‐based tools minimize inter‐observer variability and refine treatment plans, thereby reducing the risk of over‐ or under‐treatment and alleviating side effects, including radiation‐induced toxicity. Enhanced precision supports the preservation of organ function and overall health of patients.

### 4.3 Advanced Recurrence Monitoring

The capability of AI to analyze sequential imaging datasets facilitates the early identification of BCR or residual disease. This enables prompt intervention, halts disease advancement, and enhances prognosis.

## 5 DL AND AI‐BASED SEGMENTATION TECHNIQUES

The use of AI in nuclear medicine and molecular imaging represents a notable progress, fundamentally transforming both diagnostic and therapeutic methodologies. An important advancement in this field is the use of DL methods to automatically segregate soft tissue and bones in CT images. These techniques allow for accurate and fast evaluation of soft tissue and bone volumes, leading to a fundamental transformation in the field of diagnostics.^22^

The primary methods used in these approaches mainly utilize convolutional neural networks (CNNs), which are well‐recognized for their high efficiency and accuracy in image identification and analysis. CNNs are particularly advantageous in medical imaging due to their ability to learn hierarchical spatial features, enabling precise delineation of anatomical structures. Compared with other architectures, such as U‐Net, which is designed specifically for segmentation tasks, CNNs offer broader applicability and computational efficiency. Additionally, transformer‐based models, while powerful, typically require extensive datasets and higher computational resources, making CNNs a more practical choice for the current scope of nuclear medicine applications. Utilizing CNNs on CT scan data enables precise and automatic delineation of anatomical features. Segmentation plays a vital role in nuclear medicine, especially when combined with PET scans, as it enables accurate monitoring of tracer activity within these segmented skeletal areas. The integration of CT for anatomical segmentation and PET for functional imaging is particularly noteworthy in ^68^Ga‐PSMA PET‐CT studies.^23^

^68^Ga‐PSMA PET‐CT scans are increasingly used to identify and analyze the spread of PC, particularly in the skeletal system.^12^ The ^68^Ga‐PSMA tracer used in these scans has remarkable efficacy in selectively targeting PC cells, owing to its unique binding characteristics. Historically, the analysis of these scans has been mostly subjective and descriptive. Nevertheless, the emergence of AI and DL in this domain can facilitate a transition toward a more quantitative examination, providing a more intricate and all‐encompassing comprehension of the illness.

A crucial feature of AI in this field is its automated segmentation procedure. Accurate evaluation of tracer uptake in ^68^Ga‐PSMA PET‐CT images requires precise segmentation of skeletal, lymph node, and prostatic components. Manual segmentation is both time‐consuming and prone to unpredictability. However, CNN‐based automated segmentation may significantly reduce the required time and effort, resulting in more consistent and reproducible outcomes.^24^

Moreover, these developments have implications beyond the field of cancer. The same ideas of AI‐driven segmentation and quantification may be used to evaluate the metabolic processes of bones, lymph nodes, and the prostate, as well as to gauge metabolic turnover in non‐oncologic situations. This might be especially advantageous in disorders where metabolic activity plays a crucial role in the course of the disease and the response to therapy. In summary, the fusion of AI and DL with nuclear medicine is paving the way for fundamental changes in diagnostic imaging and treatment planning. This is especially apparent when these technologies are used in ^68^Ga‐PSMA PET‐CT scans, as they provide the potential for more accurate, quick, and automated analysis. This will improve the efficiency and accuracy of clinical procedures in cancer and other fields. This technical breakthrough represents a substantial progress in personalized medicine, empowering doctors to provide more customized and efficient patient treatment.^9^

## 6 RECOMIA CLOUD‐BASED AI PLATFORM IN NUCLEAR MEDICINE AND RADIOLOGY

RECOMIA, a cloud‐based annotation tool accessible at https://www.recomia.org, developed by the Research Consortium for Medical Image Analysis in Sweden, demonstrated its adaptability for highly specialized research tasks, such as quantifying the overall metabolic load in bones, lymph nodes, and the prostate during ^68^Ga‐PSMA PET‐CT. The use of RECOMIA's pre‐trained AI approaches in this specific case serves as a prime example of the platform's significant influence in the realm of nuclear medicine, particularly in the domain of PC investigation and therapy.^25^

^68^Ga‐PSMA PET‐CT is an advanced imaging technique often used for the identification and assessment of PC. It offers crucial information regarding the occurrence and magnitude of cancer metastasis, particularly in the skeletal and lymphatic systems. However, it is difficult to precisely measure the metabolic activity of cancer inside these structures, which is essential for determining the stage of the illness, devising a treatment strategy, and assessing the effectiveness of therapy.

In this study, RECOMIA was applied to a dataset comprising 50 annotated ^68^Ga‐PSMA PET‐CT scans. The dataset represented diverse clinical scenarios, including localized, nodal, and metastatic cases. Expert radiologists provided manual annotations of key anatomical regions such as the prostate gland, lymph nodes, and bones, ensuring accurate and reliable ground truth data for validation and analysis. The pre‐trained RECOMIA AI tool was then employed to perform automated segmentation and calculate the metabolic load across these regions. While manual segmentation by expert radiologists has been the gold standard in nuclear medicine imaging, the advent of AI has introduced significant advantages in terms of speed, reproducibility, and precision. To quantify the performance of the RECOMIA AI tool against expert radiologists' manual segmentations, a comparative analysis was conducted using established metrics such as the Dice similarity coefficient (DSC) and the Jaccard index. The AI‐generated segmentations achieved a mean DSC of 0.89 for prostate gland segmentation, which is nearly equivalent to the inter‐radiologist DSC agreement of 0.91. In addition, the AI tool segmented each PET‐CT scan in an average of 15 seconds, compared with the 30–45 minutes required for manual segmentation by radiologists, highlighting its significant efficiency advantage. Unlike manual methods that are subject to variability, the AI tool produced consistent results across all cases, underscoring its robustness and reliability.

The RECOMIA tool employs comprehensive data augmentation in its pre‐training phase to enhance resilience by simulating diverse imaging circumstances and artifacts. Moreover, its architecture has been engineered to accommodate fluctuations in tracer uptake patterns, patient positioning, and anatomical variability. Initial research indicates that the AI model exhibits stable performance when evaluated on datasets from several imaging centers. Nonetheless, rigorous validation studies utilizing external datasets that encompass a diverse array of clinical circumstances are essential to determine its generalizability.

RECOMIA's automated capabilities offer efficient and precise delineation of anatomical features, which facilitate advanced quantitative assessments of tumor burden. These assessments provide deeper insights into disease progression and enable the development of optimized treatment strategies. The integration of RECOMIA's AI‐driven analysis with our dataset underscores its transformative potential in the clinical workflow, enabling reproducible and highly accurate results without requiring additional model training.

This manuscript highlights the combined strength of a well‐curated proprietary dataset and the advanced capabilities of the RECOMIA tool, showcasing their utility in advancing diagnostic and therapeutic decision‐making in PC management. By using RECOMIA's pre‐trained algorithms, we achieved a high degree of accuracy and efficiency, underscoring the impact of AI in transforming nuclear medicine practices.

## 7 METHODS FOR TUMOR VOLUME ASSESSMENT WITH ^68^Ga‐PSMA PET

^68^GaPSMA‐11 PET‐CT was performed at an administered dosage of 2.2 MBq/kg of ^68^Ga‐PSMA‐11. Approximately 60 minutes after injection, imaging was performed using the Digital Biograph Vision 600, which covers the area from the vertex to the mid‐thigh. The imaging was performed in the Continuous Bed Motionmode, advancing at a rate of 1.2 mm/second. The PET used an iterative reconstruction technique that included time‐of‐flight and point‐spread function modeling. The reconstruction was performed using a matrix size of 440 × 440. Simultaneously, CT was conducted to correct the PET attenuation and provide anatomical information. CT images were obtained during the late venous phase using an adaptive statistical iterative reconstruction technique.

The scans were manually segmented using RECOMIA platform. This program includes fundamental PET‐CT visualization features and the ability to separate and analyze different image segments. The lymph node assessment adhered to the E‐PSMA criteria, which classifies nodes with grades 1–2 as non‐malignant and grades 4–5 as indicative of disease. Grade 3 nodes are considered abnormal if their uptake patterns differ from the usual non‐specific uptake, such as showing low‐to‐moderate uptake along the external iliac arteries.

A test sample consisting of 50 scans, each with three manual segmentations, was selected from the entire collection of scans. The AI tool used in this study was constructed based on the UNet3D CNN. Its purpose is to distinguish between the backdrop and lymph node metastases at the level of individual pixels. The input of the CNN consisted of a CT image, an SUV image, and a three‐channel organ mask created by an organ segmentation network. The channels in the organ mask corresponded to the prostate and urine bladder, gastrointestinal system, and bones.

The CNN was trained using image patches labeled with lymph node metastases. A categorical cross‐entropy loss function was used to minimize the disparity between the network output and human annotations using a modified version of stochastic gradient descent. The training batches consisted of eight patches of 100 × 100 × 100 pixels with a specific emphasis on lymph node metastases. The training sessions consisted of 2,500 batches for training and 500 batches for validation. To prioritize sensitivity, the annotators did not impose any penalty on background pixels that were labeled as metastatic. The training was continued for 50 epochs or until the validation loss showed no progress for 10 consecutive epochs. Subsequently, challenging instances or incorrectly labeled pixels were recognized and used in further training rounds.

The optimization process utilized the Adam optimization technique with Nesterov momentum.^26^ Strategies to mitigate overfitting included early stopping, a dropout rate of 25%, and L2 regularization.

AI models in medical imaging often face biases introduced by non‐representative datasets, potentially limiting their generalizability across diverse clinical settings. The dataset used in this study, while robust, may still reflect certain demographic or institutional biases owing to its origin from a single center. Over‐representation of specific age groups, disease stages, and imaging protocols can affect the performance of the model in broader populations. To address these biases, the dataset was augmented using advanced techniques, such as rotation, scaling, and intensity normalization, to simulate diverse imaging scenarios. In addition, the pre‐trained RECOMIA tool incorporated cross‐validation and external testing strategies to ensure consistent performance across varying clinical conditions and scanner types. Future efforts should focus on multi‐center collaborations and integration of globally sourced datasets to enhance model robustness and ensure equitable representation of different demographics and imaging protocols. During statistical analysis, lesions were classified as confirmed positives if they overlapped with the segmentation results. Lesions that did not overlap were categorized as false negatives or false positives. The sensitivity of AI was evaluated by comparing its ability to identify probable lymph node metastases with that of human readers.

## 8 CLINICAL APPLICATIONS AND FUTURE DIRECTIONS OF DL IN ^68^Ga‐PSMA PET‐CT

**FIGURE 2:**
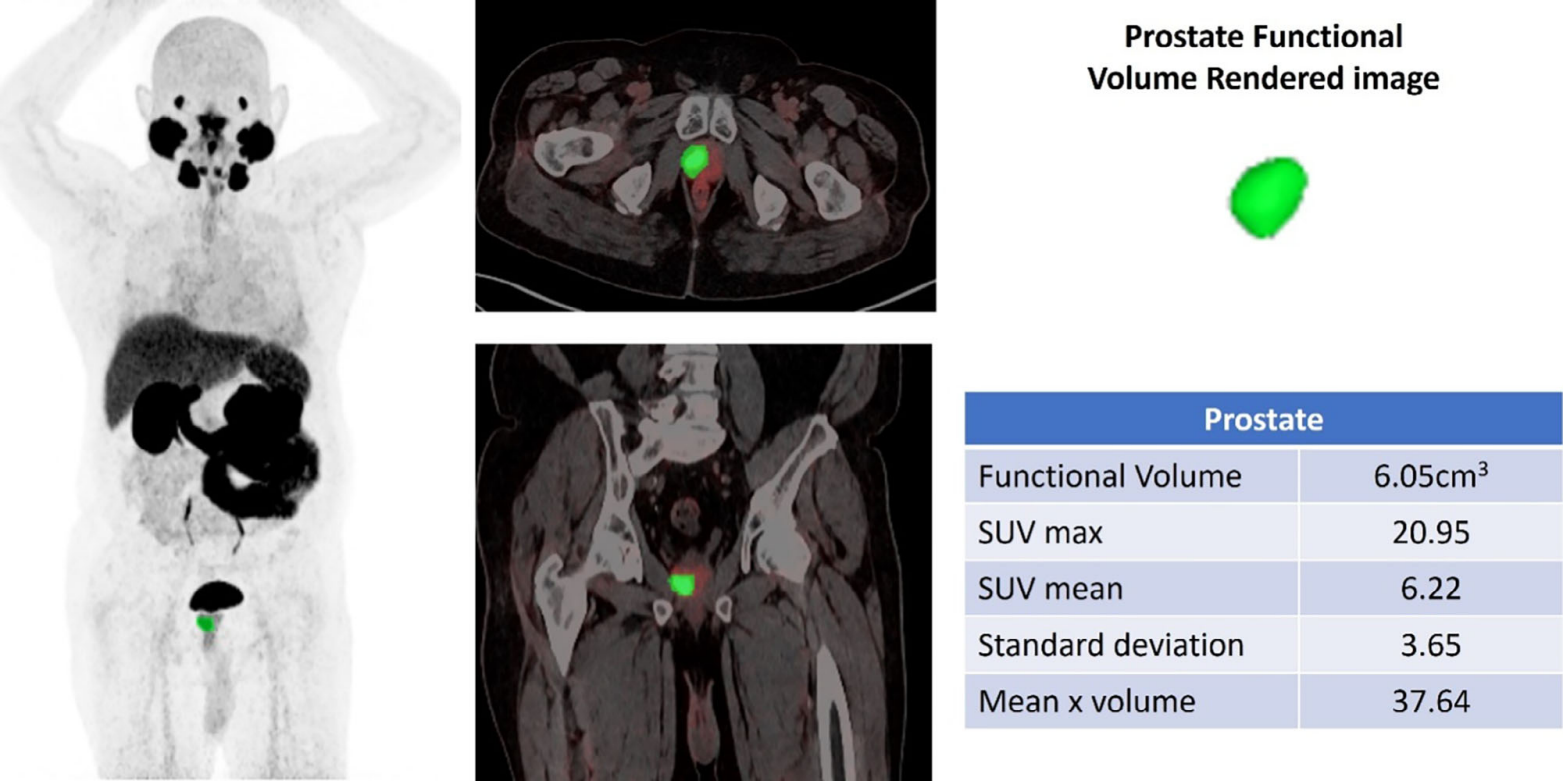
^68^Ga‐PSMA PET showing localized prostate lesion with functional volume of 6.05 cm^3^ with no evidence of distant metastasis.

**FIGURE 3:**
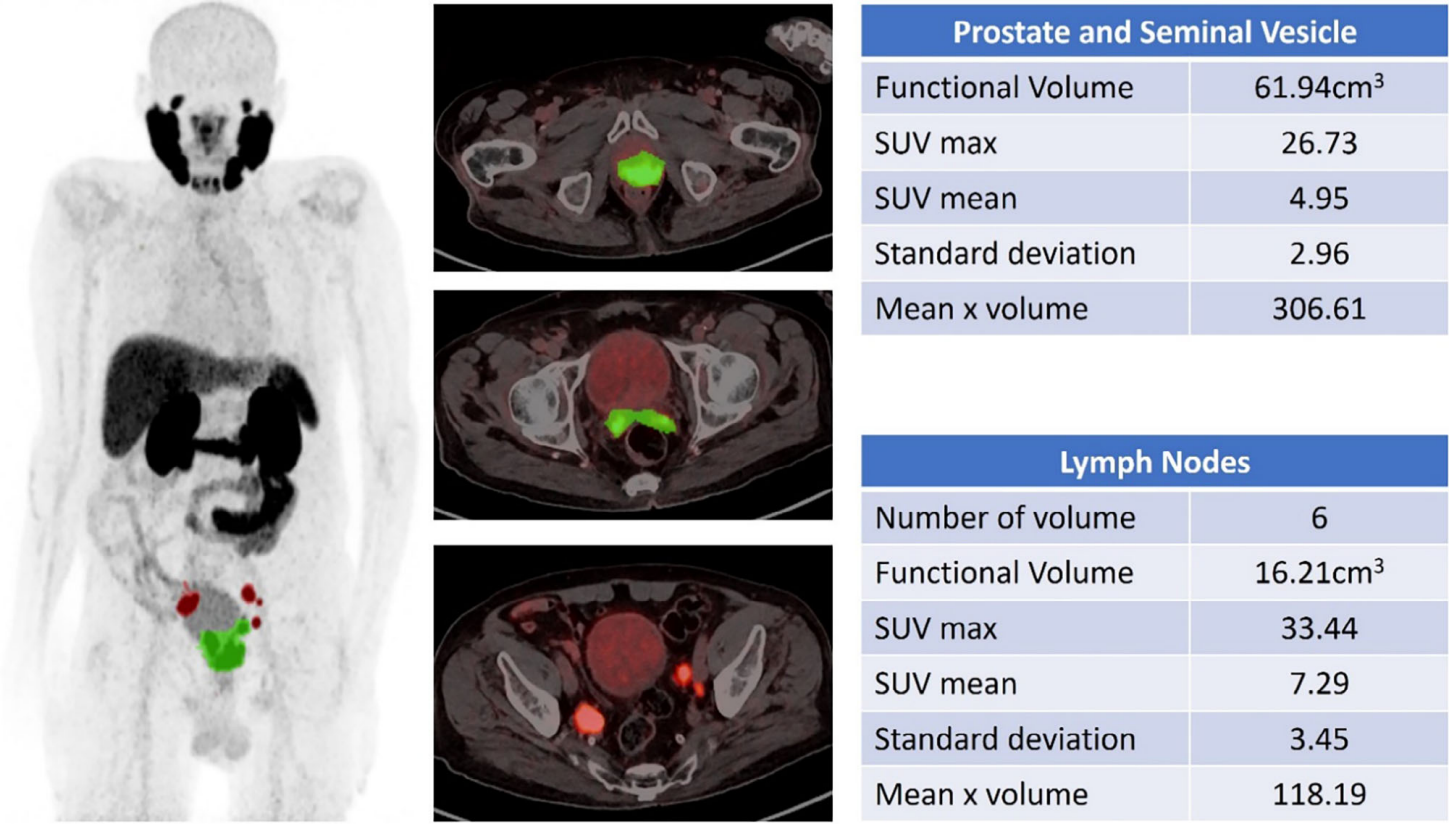
^68^Ga‐PSMA PET‐CT showing prostate lesion extending to seminal vesicle. Multiple ^68^Ga‐PSMA avid pelvic lymph nodes are observed. No evidence of distant metastasis.

**FIGURE 4:**
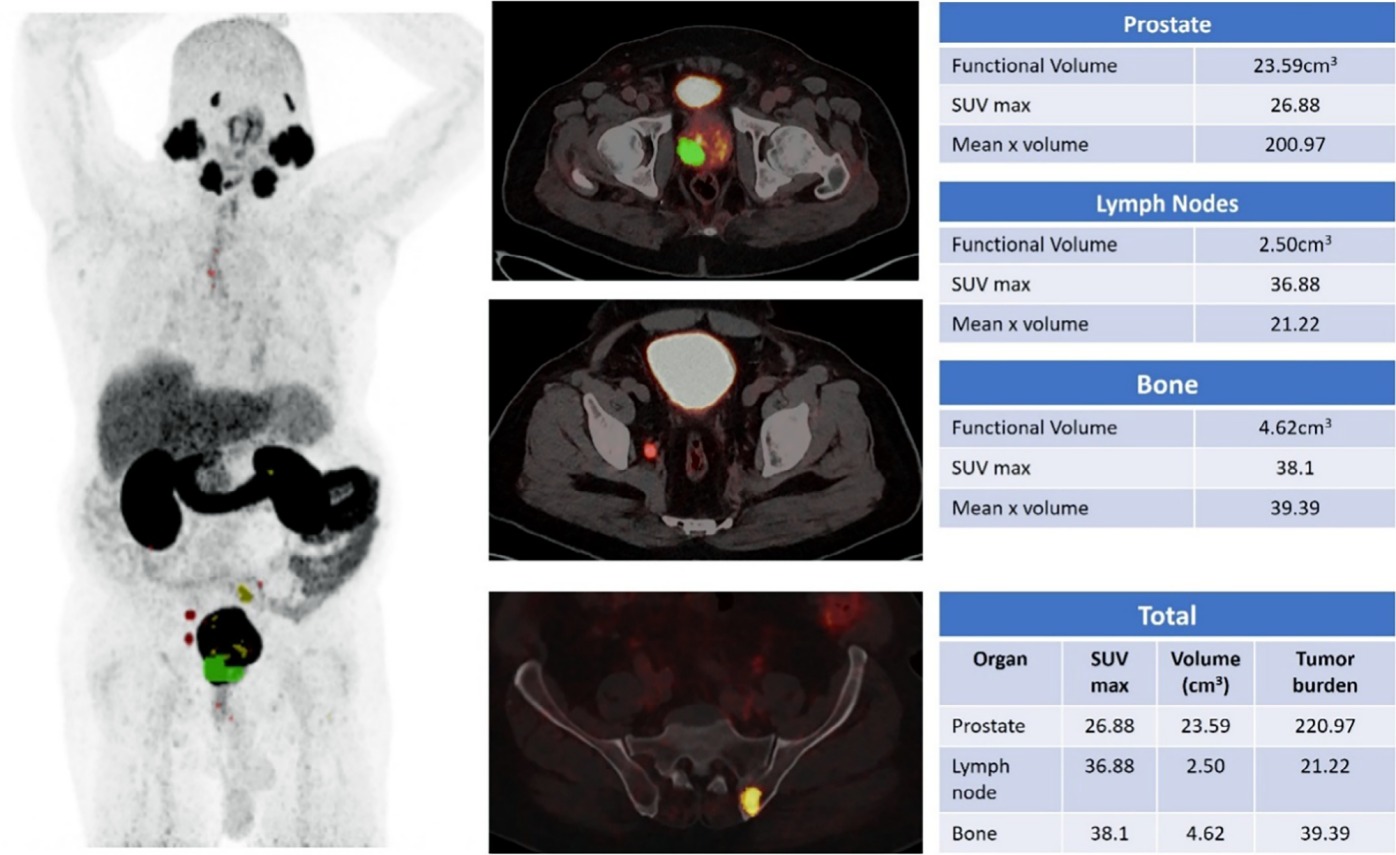
^68^Ga‐PSMA PET‐CT showing prostate lesion at right peripheral zone. Multiple ^68^Ga‐PSMA avid pelvic lymph nodes are observed and left iliac bone is consistent with metastasis.

The emergence of ^68^Ga‐PSMA PET‐CT, together with the advancing capabilities of DL analysis, offers a wide range of potential uses in medical imaging, including the diagnosis, staging, treatment planning, treatment response, and recurrence/progression of PC. The adoption of this integrated approach can completely transform several facets of oncological treatment by providing a more accurate, individualized, and anticipatory understanding. These are some of its main applications:

DL models can be used to accurately detect and analyze PC lesions in ^68^Ga‐PSMA PET‐CT images, thereby improving the ability to identify and characterize tumors (Figure 2). This feature has the potential to facilitate early identification, particularly when conventional imaging techniques are inadequate. DL facilitates precise staging of PC using quantitative analysis to measure the degree of tumor dissemination, namely to bones and lymph nodes, which are frequent locations of metastasis (Figures 3 and 4). The accuracy of the staging process is crucial for selecting the most efficient treatment strategy.

### 8.1 Diagnosis and Treatment Planning Guidance

Precise mapping of disease using DL analysis of ^68^Ga‐PSMA PET‐CT images may provide surgeons and radiation oncologists with valuable guidance in devising treatment strategies. For example, it may aid in directing biopsy needles toward the most significant regions for better cancer detection, and accurate Gleason Score assessment, which in turn help in deciding the best treatment option and defining the specific area to be targeted for radiation treatment, especially additional boost doses of radiation to overcome bulky lesions.^27^

### 8.2 Assessing Treatment Response

Consistent use of advanced DL analysis on ^68^Ga‐PSMA PET‐CT images over the course of treatment may provide a comprehensive understanding of the reaction of cancer to therapy. This information may be vital for making prompt choices regarding whether to change or continue the existing treatment plan.

#### 8.2.1 Prediction of Treatment results

AI algorithms can forecast treatment results by analyzing ^68^Ga‐PSMA scans, both initially and continuously. By examining trends and results from past instances, these models can predict the probability of success for certain treatment methods.

#### 8.2.2 Personalized Therapy Recommendations

DL analysis may aid in creating customized treatment regimens by considering the distinct attributes of the tumor, as well as the patient's individual medical history. Customizing treatment techniques can enhance effectiveness and reduce adverse effects.

The use of DL analysis in conjunction with ^68^Ga‐PSMA PET‐CT enhances sensitivity, allowing for timely identification of cancer recurrence. This is of utmost importance for the long‐term management of PC.

### 8.3 Research and clinical trials for utilizing DL

DL analysis of ^68^Ga‐PSMA PET‐CT scans in research settings may effectively categorize individuals for clinical trials, ensuring that medicines are evaluated in the most suitable patient cohorts. By utilizing DL algorithms, it is possible to identify new biomarkers from ^68^Ga‐PSMA PET‐CT scan data. These biomarkers have the potential to play a crucial role in understanding the advancement of diseases and effectiveness of treatments. In the future, advancement scan be made by combining genetic information with imaging data. This involves the use of DL analysis of ^68^Ga‐PSMA images, together with genomic data, to gain a more complete understanding of the disease.

The prospective use of DL analysis in ^68^Ga‐PSMA PET‐CT imaging is extensive and encouraging. The integration of this technology has the potential to result in substantial advancements in the identification, categorization, therapy, and tracking of PC, eventually leading to enhanced patient outcomes and more effective healthcare provisions. The advancement of AI technology will inevitably lead to an expansion of its function in increasing the capabilities of sophisticated imaging methods, such as ^68^Ga‐PSMA PET‐CT. This will provide new opportunities for personalized medicine and cancer treatments.

## 9 CHALLENGES AND PROSPECTS IN THE ANALYSIS OF ^68^Ga‐PSMA USING DL

Although there are several potential uses of DL in ^68^Ga‐PSMA PET‐CT, there are significant challenges that need to be resolved. Overcoming these challenges is essential for fully exploiting the advantages of this technology in clinical practice. The following are the significant challenges and potential prospects for future development in this domain.

### 9.1 Data Availability and Quality

The availability of high‐quality and varied datasets is crucial for training and validating DL models. Large datasets may have constraints in terms of availability, particularly regarding standardized labeling and complete clinical information. Ensuring the robustness and validity of AI models across diverse patient groups and imaging equipment is a significant challenge in terms of model and validation. Extensive validation is often required for models to demonstrate their efficacy and dependability in many clinical environments.

### 9.2 Understanding AI Decisions

DL models, particularly those employing complex architectures, may operate as “opaque systems,” creating difficulties in understanding the reasoning behind their outcomes. This lack of interpretability may impede the adoption of this method in clinical settings.

To resolve this issue, explainable AI (XAI) techniques can be used to elucidate the decision‐making processes of AI models, thereby improving their transparency and reliability. Gradient‐weighted class activation mapping (Grad‐CAM) emphasizes the areas in an input image that substantially affect a model's predictions. When utilized with ^68^Ga‐PSMA PET‐CT images, Grad‐CAM facilitates the visualization of regions within lymph nodes or bones that the model designates as indicative of metastasis, allowing doctors to ascertain whether AI concentrates on clinically pertinent aspects. Similarly, SHapley Additive exPlanations (SHAP) can assess the contribution of specific features, such as SUV measurements or anatomical segmentations, to the predictions made by AI. These strategies enhance the interpretability of AI models and enable doctors to evaluate their conformity with recognized medical knowledge.

The incorporation of AI‐based segmentation into clinical practice requires specific steps to ensure seamless adoption and minimal disruption. Workflow automation, compatibility with DICOM standards, and intuitive user interfaces are critical for smooth integration. Validation through phased studies and feedback loops can build trust, while training clinicians on tools such as RECOMIA, ensures effective utilization. Additionally, explainability techniques such as Grad‐CAM and SHAP embedded in clinical workflows can foster confidence in AI‐driven imaging, supporting informed decision‐making. Incorporating AI functionalities into existing healthcare systems without disrupting them is challenging. Integrating XAI approaches into tools such as RECOMIA via intuitive interfaces will facilitate clinical uptake, guaranteeing that models augment rather than hinder clinical efficiency. Subsequent research should emphasize the integration of interpretability methodologies into clinical workflows to enhance confidence and trust in AI‐Driven medical imaging.

### 9.3 Failure Modes in AI‐Based Segmentation

Despite these advantages, AI‐based segmentation models have limitations in specific scenarios. For example, segmenting tumors with irregular shapes, low contrast, or close proximity to healthy tissues can challenge even advanced models. AI may also struggle with tumors located in uncommon anatomical regions or with atypical tracer uptake patterns, leading to false negatives or suboptimal contours. These failure modes can directly affect treatment planning, particularly in radiotherapy, where inaccuracies in tumor volume delineation may result in either underdosing of the tumor or overdosing of healthy tissues.

To address these limitations, combining AI segmentation with human expertise can improve results. Incorporating a feedback mechanism through which clinicians can review and refine AI‐generated contours enhances both accuracy and trust. Future model training should prioritize incorporation of more diverse datasets representing various tumor shapes, tracer patterns, and anatomical variations to mitigate these challenges.

### 9.4 Regulatory and Ethical Considerations

Navigating the regulatory framework for AI in healthcare is a complex task. The U.S. Food and Drug Administration and European Conformity (CE) frameworks mandate that AI‐based medical devices adhere to rigorous safety, efficacy, and reliability requirements. In the United States, AI tools must traverse approval processes such as 510(k) clearance, De Novo classification, or premarket approval, facing specific hurdles for continually learning AI models that may necessitate additional reviews or re‐approvals for algorithm modifications. In Europe, the Medical Device Regulation regulates CE marking for AI tools, highlighting the importance of clinical evidence, risk management, and transparency in decision‐making processes.

Moreover, ethical considerations such as securing patient consent and safeguarding data privacy are paramount. Adherence to standards, such as the General Data Protection Regulation (GDPR) in Europe, is essential for preserving patient trust. In addition, mitigating biases in model training by integrating various datasets is crucial for fostering equal access to AI tools. Establishing frameworks that emphasize transparency, explainability, and inclusion is essential for addressing these difficulties and cultivating trust in AI‐driven medical imaging systems.

This study meticulously managed patient data to ensure adherence to all regulations. All imaging data utilized for model validation were anonymized by eliminating personal information, such as names, dates of birth, and medical record numbers, before being submitted to the RECOMIA platform. The anonymization procedure adhered to the GDPR, safeguarding patient privacy during the study.

Robust data storage and transmission techniques were used to protect sensitive information. Encrypted communication routes facilitated data uploads, and the RECOMIA platform has stringent cybersecurity protocols, encompassing restricted access and audit trails. Furthermore, informed consent was obtained from all participants, specifying the use of anonymized data for research objectives.

### 9.5 Cost and Resource Allocation

The development and implementation of AI systems require substantial resources. Healthcare practitioners must meticulously assess the costs related to potential therapeutic benefits as a critical consideration. AI segmentation systems require initial expenditures for software licensing, integration, and personnel training, although they can yield significant long‐term savings by enhancing workflow efficiency and decreasing treatment planning duration. AI can substantially reduce the time required for tumor delineation, enabling physicians to concentrate on intricate situations and perhaps lowering labor expenses. The enhanced accuracy of AI‐generated segmentation can facilitate more focused medicines, reducing the costs associated with treatment‐related toxicities and improving patient outcomes.

A cost‐benefit analysis comparing AI with manual approaches underscores the possibility of net savings, regardless of initial expenditures. Shorter planning duration and less variability in tumor delineation can improve clinical efficiency, resulting in financial advantages for healthcare organizations. Moreover, employing AI techniques in adaptive radiotherapy workflows may enhance resource efficiency by guaranteeing precise treatment plans without the need for recurrent manual modifications. Although the initial financial outlay may be considerable, the enduring therapeutic and operational advantages frequently validate expenditures, rendering AI a beneficial enhancement for contemporary clinical procedures.

## 10 MANAGING UNCERTAINTY IN AI PREDICTIONS

A crucial element of AI in medical imaging is its capacity to measure uncertainty in its predictions, which directly influences the reliability and interpretability of the outcomes. This study addressed uncertainty quantification using methodologies aimed at assessing confidence levels in AI‐generated segmentations. The DL‐based model utilized a probabilistic framework to produce segmentation outputs by attributing confidence values to each pixel or voxel within the segmented areas. This facilitated the identification of locations where model predictions exhibited diminished certainty, such as areas with poor contrast or uncertain anatomical delineations.

### 10.1 Threshold‐Based Refinements

Confidence thresholds were applied to the probabilistic outputs to ascertain the final segmentations. Regions of low confidence were designated for manual assessment by physicians, ensuring that the model's predictions were augmented with expert validation in ambiguous circumstances.

### 10.2 Monte Carlo Dropout

A dropout method was employed during inference to replicate the heterogeneity in the model's predictions. By producing numerous segmentation samples with identical inputs and examining their variability, the model assessed the epistemic uncertainty (uncertainty arising from model constraints). These data were utilized to enhance segmentation reliability by concentrating on areas with consistent predictions.

### 10.3 Visual Feedback for Clinicians

Uncertainty maps were superimposed on the original imaging data to graphically denote regions of high and low confidence. This input enabled clinicians to identify areas that require enhanced examination or manual modifications.

Measuring uncertainty in AI predictions enhances the robustness of results and fosters trust by allowing physicians to comprehend and respond to a model's confidence level. Future initiatives should prioritize the incorporation of sophisticated uncertainty estimation methodologies, such as Bayesian DL, into clinical workflows to augment the interpretability and dependability of AI instruments in PC imaging.

## 11 FUTURE PROSPECTS

DL for PC imaging with ^68^Ga‐PSMA PET holds great promise for the future, particularly for tumor volume assessment using AI‐based segmentation.

### 11.1 Standardizing and Sharing High‐Quality Imaging Datasets

Standardized datasets are essential for improving data collection and sharing. Cooperative endeavors, perhaps on a worldwide scale, may greatly bolster the advancement of resilient AI models.

### 11.2 Enhancing Interpretability

Improving the interpretability of AI models will boost confidence and usefulness in healthcare situations, ensuring that the models are not only accurate but also understandable. XAI is expanding and has the potential to address this problem.

### 11.3 Collaboration

Continued cooperation among AI researchers, radiologists, oncologists, and other stakeholders is essential for developing clinically relevant and efficacious treatments.

### 11.4 Personalized Treatment Regimens

AI holds promise in developing personalized treatment regimens based on specific patient features and illness profiles.

### 11.5 Integration of Multimodal Data

Combining imaging data with genetic, proteomic, and clinical data provides a comprehensive understanding of cancer and its treatment, thereby facilitating the development of precision medicine.

### 11.6 Stringent Validation and Regulation

Creating stringent validation processes and well‐defined regulatory channels is crucial for introducing AI solutions into clinical settings. This entails the establishment of criteria for evaluating the performance and safety of AI models.

### 11.7 Cost‐Effectiveness Studies

Conducting cost‐effectiveness studies to assess the economic efficiency of AI applications in healthcare contexts is crucial for promoting their broader use.

### 11.8 Education and Training

Providing healthcare workers with knowledge and understanding of the potential and limitations of AI is crucial. This includes instructions for the efficient integration of AI technologies into clinical practice.

### 11.9 Transfer Learning for Low‐Data Scenarios

Leveraging transfer learning techniques can address the challenges posed by limited annotated datasets in ^68^Ga‐PSMA PET. By utilizing pre‐trained models developed on larger datasets from related domains, transfer learning can accelerate model training, enhance segmentation accuracy, and improve generalizability. Future studies should explore the application of transfer learning to optimize AI performance in PC imaging, particularly in centers with limited imaging data.

To fully utilize the capabilities of DL in ^68^Ga‐PSMA PET/CT imaging, it is essential to address these problems and pursue these future directions. As this discipline progresses, it offers significant potential for improving the diagnosis and management of PC, eventually resulting in improved patient care and outcomes.

## 12 VALIDATION THROUGH CLINICAL TRIALS

To validate the clinical utility of AI‐driven segmentation in **^68^Ga‐PSMA PET‐CT**, several ongoing and proposed clinical trials aim to assess its impact on treatment outcomes, workflow efficiency, and patient quality of life. These trials are critical for establishing evidence‐based practices to integrate AI into PC imaging.

### 12.1 Proposed Clinical Trials

Future trials should focus on evaluating how AI‐enhanced segmentation influences radiotherapy planning, particularly in reducing inter‐observer variability and improving dose delivery accuracy. Trials designed to compare AI‐assisted workflows with standard manual approaches can quantify the time saved, toxicity reduction, and survival benefits.

### 12.2 Ongoing Trials

- **NCT04457245**: This trial investigates the utility of ^68^Ga‐PSMA PET‐CT in the staging and management of high‐risk patients with PC, with an emphasis on integrating AI to improve imaging accuracy.^28^
- **NCT04851178**: Evaluates the role of AI in detecting BCR using PSMA‐targeted imaging to provide quantitative evidence of its diagnostic accuracy and clinical impact.^29^
- **NCT05188933**: Focuses on the role of AI‐driven imaging tools in predicting patient outcomes and personalizing radiotherapy plans for advanced PC.^30^

These trials will provide robust data on the performance of AI‐enhanced ^68^Ga‐PSMA PET in diverse clinical scenarios, helping refine treatment strategies and ensure its adoption in broader clinical workflows.

## 13 CONCLUSIONS

The assessment of regional and total tumor volumes with ^68^Ga‐PSMA PET using AI‐based segmentation represents a significant advancement in PC imaging. AI algorithms offer several advantages over traditional manual segmentation methods including improved accuracy, reproducibility, and efficiency. The integration of AI‐based segmentation into clinical practice could potentially enhance treatment planning, response assessment, and prognostication in PC patients. However, several challenges remain, such as the need for standardized protocols, validation studies, and seamless integration with clinical workflows. Future research should address these challenges and further optimize AI algorithms for ^68^Ga‐PSMA PET. In addition, efforts to establish consensus guidelines and regulatory frameworks for the use of AI in medical imaging are essential for its widespread adoption and clinical utility. Overall, AI‐based segmentation holds great promise for advancing the field of PC imaging and improving patient care.

## CONFLICT OF INTEREST STATEMENT

There are no potential conflicts of interest to disclose.

## ETHICS STATEMENT

Not applicable.

## Data Availability

Data sharing not applicable to this article as no datasets were generated or analysed during the current study
